# Survival in patients with CKD 3–5 after 12 months of exercise training – a post-hoc analysis of the RENEXC trial

**DOI:** 10.1186/s12882-024-03915-1

**Published:** 2025-01-23

**Authors:** Sara Denguir, Matthias Hellberg, Martin Almquist, Naomi Clyne

**Affiliations:** 1 Department of Nephrology, Department of Clinical Sciences Lund, Nephrology, Faculty of Medicine, Skåne University Hospital and Lund University, Lund, Sweden; 2 Department of Surgery, Department of Clinical Sciences Lund, Surgery, Faculty of Medicine, Skåne University Hospital and Lund University, Lund, Sweden

**Keywords:** Chronic kidney disease, Physical performance, Physical activity, Exercise training, 6-Minute walking test, Sit-To-Stand test, Handgrip test, Mortality, Survival

## Abstract

**Background:**

Physical performance is low and physical activity declines in people with chronic kidney disease (CKD). Both factors are associated with decreased survival. Our hypothesis was that improved physical performance after 12 months of exercise training would result in better survival in patients with CKD stages 3 to 5 not on kidney replacement therapy (KRT). Our aims in this study were to investigate the survival effects of (1) baseline physical performance and (2) physical performance after 12 months of exercise training.

**Methods:**

This is a post-hoc analysis of the RENEXC trial, a randomized controlled study comparing 12 months of strength- and balance training both in combination with aerobic training. Both groups improved physical performance with no between group differences. Patients were categorized into five groups: improved *≥* 5%, unchanged, deteriorated *≥* 5%, non-completers, missing data. Univariate and multivariate Cox regression analyses were used and adjusted for age, sex, comorbidity, time on dialysis and time with a kidney transplant.

**Results:**

151 patients participated, mean age 66 *±* 14 years, 65% men, eGFR 22.5 *±* 8.2 ml/min/1.73m^2^, average follow-up 60 months.

**Multivariate analyses:**

The baseline 6-minute walk test (6MWT) (HR 0.996; 95% CI [0.993–0.998]) and 30-second sit-to-stand (30s-STS) (HR 0.94 CI [0.89–1.0]) were positively associated with survival. After 12 months of exercise improved handgrip strength (HGS) right (HR 2.66; 95% CI [1.07–6.59]) was associated with better survival compared with deterioration. Improvement compared with noncompletion was associated with better survival (6MWT (HR 2.88; 95% CI [1.4–5.88]), HGS right (HR 4.44; 95% CI [1.79–10.98]), functional reach (HR 3.69; 95% CI [1.82–7.48]), isometric quadriceps strength right (HR 2.86; 95% CI [1.43–5.72]), 30s-STS (HR 3.44; 95% CI [1.66–7.11]).

**Conclusion:**

Baseline walking distance, muscular strength and endurance in the legs were independently associated with survival in people with CKD stages 3–5 without KRT. After completing 12 months of exercise training improved walking distance, muscular strength and endurance, and balance were positively associated with survival, compared with noncompleters. Better physical performance at baseline and the ability to complete 12 months of exercise training conferred survival benefits. There are probably several factors affecting better survival. These factors require elucidation in future studies.

**Trial registration:**

ClinicalTrials.gov NCT02041156. Registration date 20,240,107.

## Background

Patients with chronic kidney disease (CKD) have impaired physical performance [1–3] and are generally less physically active compared with the general population [3–7]. Both these factors are associated with decreased survival [8–18].

Earlier studies have shown that patients with CKD can improve their physical performance with exercise [19–22] and that exercise has health-promoting effects such as reduction of left ventricular hypertrophy, fewer arrythmias, better blood pressure, reduced arterial stiffness, less inflammation and improved quality of life [23–28].

Recently, a meta-analysis showed that a higher level of self-reported physical activity was associated with better survival in people on kidney replacement therapy including dialysis and transplantation (KRT) [29]. In another meta-analysis measured physical performance showed a positive association with survival in people with CKD stages 2–5 and on dialysis [30]. A previous study in patients at all stages of CKD as well as on KRT reported that patients who participated in a renal rehabilitation program and improved their walking distance after 12 weeks of exercise had a lower risk of the combined outcome of cardiovascular disease and mortality compared with patients who did not improve [31]. The RENEXC (RENal EXCercise) trial is, to date, the largest exercise training trial in people with CKD not on KRT with a duration of 12 months. The effect of participation in a long-term exercise training program on survival alone has, to our knowledge, not been studied in this patient population. In this post-hoc analysis of the RENEXC trial we studied survival in patients with CKD stages 3–5 not on KRT, who participated in exercise training [21].

## Methods

### Hypothesis and aims

Our hypothesis was that improved physical performance after exercise training would result in better survival. Our aims were to investigate the survival effects of (1) baseline physical performance and (2) physical performance after 12 months of exercise training.

### Study design and setting

This study is a post-hoc observational follow-up study of the randomized, controlled exercise training trial, RENEXC, which was conducted at the Department of Nephrology at the University Hospital of Skåne in Lund between 2011 and 2017. In this study all data were pooled as there were no statistically significant between group differences for the two randomization arms for any of the measures of physical performance after the 12-months period of intervention. Consequently, in this study all patients from the original two arms of the RENEXC trial are treated as one cohort. Detailed information of the RENEXC trial is given in the primary report [21]. For clarity some information is repeated here. The RENEXC trial compared the effects of two training regimens: strength training and balance training, both in combination with endurance training comprising adult patients of all ages, with and without co-morbidities but not on KRT. Starting point of the present study was defined as the end of the 12 months’ exercise training intervention. Patients were followed until death, lost-to-follow-up or end-of-follow-up on 31 October 2019. The primary outcome was death. Follow-up data including start on dialysis, transplantation, mortality and laboratory data were obtained from patient records.

### Characteristics of participants

A total of 217 patients were screened and 151 were included, 98 men (65%). The mean age was 66 *±* 14 years, range 19–87, the mean eGFR was 22.5 *±* 8.2 ml/min/1.73m^2^. Three patients were unable to perform baseline measurements for medical reasons and did not partake in the intervention. One patient was lost to follow-up a few years after inclusion. 112 patients completed 12 months of exercise training. Median follow-up was 60 months (interquartile range 44–78 months). During this period 60 patients (40%) started dialysis and 18 (12%) were transplanted. 61 patients (40%) had died by the end of follow-up, of these four patients died within 1 year of inclusion. Some baseline clinical characteristics are presented in Table 1.

**Table 1:** Some baseline clinical characteristics

Variable	*n* = 151	Mean *±* SD or 25th – 50th – 75th percentile or frequency and percentage
Age (years)	151	66 *±* 14
Sex Male Female		98 (65%) 53 (35%)
Weight (kg)	151	81 *±* 18
Height (cm)	148	172 *±* 9
CKD stage CKD 3 CKD 4 CKD 5		30 (20%) 94 (62%) 27 (18%)
CRP (mg/L)	151	1.4–3.0–6.2
Hemoglobin (g/L)	150	126 *±* 14
Creatinine (µmol/L)	151	256 *±* 102
Urea (mmol/L)	151	16 *±* 5
Albumin (g/L)	150	37 *±* 4
24 h systolic blood pressure (mmHg)	133	130 *±* 15
24 h diastolic blood pressure (mmHg)	133	75 *±* 10
Davies score 0 1 2		18 (12%) 93 (62%) 40 (26%)

Abbreviations: Chronic kidney disease (CKD)

### Exercise training

Patients were prescribed an individualized exercise training program consisting of 60 min of endurance training combined with 90 min of either strength- or balance training per week. The training program was self-administered at home or at a local gym and monitored by the research physiotherapist. The intervention period lasted for 12 months. A detailed description is given in the primary report [21].

### Tests of physical performance

Different aspects of physical performance were evaluated with a comprehensive battery of tests at start and after 4, 8 and 12 months. Below is a brief description of the tests which have been described in more detail previously [1].

**The 6-minute walk test (6MWT)** was used to assess aerobic endurance. Patients were instructed to walk as fast as possible on a flat surface, back and forth between 2 cones placed 30 m apart for 6 min. The total walking distance in meters was registered.

**Handgrip strength** was measured in kilograms (kg) with a Jamar dynamometer and used to evaluate distal muscle strength in the forearm. The average of 3 measurements for each hand was registered in kg.

**Isometric quadriceps strength (IQS)** was used to assess proximal muscle endurance and strength in the legs. Patients sat on a bench with parallel thighs, flexed knees, feet hanging freely from the floor and with a dynamometer-cuff attached to the ankle. The patient was asked to extend their leg as much as possible and the distance between the knee joint and the cuff was measured in centimeters (cm). The test was repeated 3 times for each leg and the average quadriceps strength was recorded in (kg x cm).

The **30-seconds sit-to stand (30s-STS)** test was used to evaluate muscular endurance and strength in the legs by assessing how many times a patient could rise from a chair in 30 s.

**Functional reach** was used to assess balance by measuring how far patients could reach forward, standing next to a wall without leaning against it or losing balance. The average of 3 trials was recorded.

### Comorbidity

Davies comorbidity index was used to determine comorbid burden. Presence of the following comorbidities: malignancy, ischemic heart disease, peripheral vascular disease, diabetes mellitus, left ventricular dysfunction, systemic collagen vascular disease or other significant of pathology was assessed by the same physician (M.H.) at baseline [32]. 

### Statistical analysis

Normally distributed, continuous data are presented as means ± standard deviation (SD) and non-normally distributed data are presented as medians with interquartile ranges. Categorical data are given as frequencies and percentages. Survival analyses were calculated with univariate and multivariate Cox regression analyses performed in the statistical software SPSS version 26. Multivariate analyses were adjusted for age, sex, comorbidity, eGFR (estimated with the modification of diet in renal disease [MDRD] formula), time on dialysis and time with a functioning transplant. Physical performance at baseline was used as a continuous variable. Participants with missing data were excluded from the analyses with physical performance at baseline. Patients were then divided into 5 groups based on change in physical performance in each test after 12 months. The first group consisted of patients who improved their physical performance by *≥* 5%, the second of patients who maintained their physical performance, defined as less than 5% change; the third of patients who deteriorated by *≥* 5%; the fourth of patients who did not complete 12 months of exercise and the fifth of patients with missing data. Patients who died within one year of inclusion were excluded from the group that did not complete the study. A change of 5% was chosen to ensure that there was a real change in physical performance. A confidence interval (CI) of 95% was considered significant. A p value < 0.05 was considered significant.

## Results

The physical performance results have been presented previously [21], but are shown in Table 2 for clarity.

**Table 2:** Measures of physical performance at baseline and after 12 months of exercise training

	*n*	Baseline	12 months	*P*
6-minute walk test, m	100	417 ± 125	460 ± 130	< 0.001
Handgrip strength right, kg	112	32.4 ± 10.8	32.8 ± 11.3	0.3
Handgrip strength left, kg	112	29.9 ± 11.0	30.1 ± 11.0	0.6
Functional reach, cm	107	33.2 ± 8.1	35.8 ± 7.5	< 0.001
Isometric quadriceps strength right, kgxm	106	11.6 ± 3.9	12.7 ± 4.3	< 0.001
Isometric quadriceps strength left, kgxm	106	11.4 ± 4.2	12.5 ± 4.7	< 0.001
30-seconds sit-to-stand, n	104	11.4 ± 5.7	12.8 ± 7.4	0.0001

### Association of physical performance at baseline with survival

Results from the Cox regression analyses with measures of physical performance at baseline are presented in Table 3.

**Table 3:** Risk of death in relation to baseline measures of physical performance using Cox regression analyses

	Univariate analysis		Multivariate analysis	
	HR	95% CI	HR	95% CI
6-minute walk test, m	**0.995**	**0.993–0.996**	**0.996**	**0.993–0.998**
Handgrip strength right, kg	**0.971**	**0.948–0.994**	0.965	0.931–1.000
Handgrip strength left, kg	0.983	0.959–1.007	0.989	0.954–1.024
Functional reach, cm	**0.936**	**0.911–0.962**	0.970	0.933–1.008
Isometric quadriceps strength right, kgxm	0.999	0.999-1.000	1.000	0.999–1.001
Isometric quadriceps strength left, kgxm	0.999	0.999-1.000	1.000	0.999–1.001
30-seconds sit-to-stand, n	**0.892**	**0.853–0.934**	**0.943**	**0.893–0.995**

Multivariate Cox regression analyses are adjusted for age, sex, comorbidity (Davies score), eGFR, time on dialysis and time as transplanted. Abbreviations: Hazard ratio (HR), Confidence interval (CI). Estimated glomerular filtration rate (eGFR)

### Univariate Cox regression analyses

Physical performance at baseline showed a significantly positive association with survival measured as walking distance with 6MWT (HR 0.995; 95% CI [0.993–0.996]), handgrip strength right (HR 0.97; 95% CI [0.95–0.99]), balance measured with functional reach (HR 0.94; 95% CI [0.91–0.96]) and muscular strength and endurance measured with 30s-STS (HR 0.89; 95% CI [0.85–0.93]).

### Multivariate Cox regression analyses

After adjustment for age, sex, comorbidity, eGFR, time on dialysis and time as transplanted the 6MWT (HR 0.996; 95% CI [0.993–0.998]) and the 30s-STS (HR 0.94 CI [0.89–0.995]) were significantly associated with better survival.

### Association of physical performance after 12 months of exercise training with survival

Results from the Cox regression analyses with measures of physical performance after 12 months of exercise training are presented in Table 4. Creating subgroups from the relatively small sample size may affect statistical validity, but functions as a sensitivity analysis of our main hypothesis that exercise affects survival.

**Table 4:** Risk of death in relation to change in physical performance using Cox regression analyses

		Univariate analysis		Multivariate analysis	
	*n*	HR	95% CI	HR	95% CI
**6-minute walk test** Improved *≥* 5% Missing Not completed Deteriorated *≥* 5% No change	56 12 35 15 29	1 **3.369** **2.157** **2.974** 0.850	**1.424–7.971** **1.076–4.324** **1.335–6.626** 0.346–2.085	1 1.802 **2.875** 1.280 0.642	0.719–4.519 **1.405–5.884** 0.550–2.981 0.249–1.654
**Handgrip strength right** Improved *≥* 5% Missing Not completed Deteriorated *≥* 5% No change	41 1 35 32 38	1 4.941 **3.128** **3.630** 1.895	0.615–39.666 **1.348–7.260** **1.577–8.360** 0.784–4.577	1 **16.973** **4.436** **2.655** 1.288	**1.745–165.120** **1.792–10.981** **1.070–6.591** 0.519–3.195
**Handgrip strength left** Improved *≥* 5% Missing Not completed Deteriorated *≥* 5% No change	35 0 35 39 38	1 - **4.187** **4.949** 2.267	- **1.543–11.357** **1.881–13.021** 0.798–6.442	1 - **5.124** **2.990** 1.330	- **1.832–14.333** **1.108–8.070** 0.452–3.911
**Functional reach** Improved *≥* 5% Missing Not completed Deteriorated *≥* 5% No change	59 5 35 23 25	1 **11.251** 1.808 0.944 1.280	**3.667–34.521** 0.943–3.469 0.398–2.240 0.579–2.832	1 **9.601** **3.690** 1.492 1.866	**2.927–31.490** **1.821–7.475** 0.590–3.777 0.828–4.207
**Isometric quadriceps strength right** Improved *≥* 5% Missing Not completed Deteriorated *≥* 5% No change	62 7 35 27 16	1 2.857 1.733 0.916 1.428	0.974–8.381 0.913–3.291 0.406–2.069 0.605–3.369	1 2.134 **2.861** 0.954 1.197	0.701–6.501 **1.430–5.722** 0.409–2.229 0.489–2.933
**Isometric quadriceps strength left** Improved *≥* 5% Missing Not completed Deteriorated *≥* 5% No change	64 6 35 21 21	1 **5.837** **2.235** 2.040 1.638	**1.917–17.771** **1.139–4.385** 0.933–4.459 0.728–3.685	1 2.713 **3.615** 1.800 1.574	0.841–8.752 **1.747–7.477** 0.768–4.221 0.670–3.696
**30-seconds sit-to-stand** Improved *≥* 5% Missing Not completed Deteriorated *≥* 5% No change	67 9 35 26 10	1 **3.994** **2.390** **2.344** 2.103	**1.465–10.891** **1.218–4.689** **1.137–4.831** 0.775–5.710	1 **4.254** **3.438** 1.437 1.246	**1.439–12.574** **1.664–7.106** 0.650–3.175 0.429–3.622

Multivariate Cox regression analyses are adjusted for age, sex, comorbidity (Davies score), eGFR, time in dialysis and time as transplanted. Abbreviations: Hazard ratio (HR), Confidence interval (CI). Estimated glomerular filtration rate (eGFR)

### Univariate Cox regression analyses

Patients who improved their walking distance, handgrip strength, muscular strength and endurance by 5% or more after 12 months of exercise had a significantly better survival compared with patients who deteriorated by at least 5% (6MWT (HR 2.97; 95% CI [1.34–6.63]), handgrip strength right (HR 3.63; 95% CI [1.58–8.36]), handgrip strength left (HR 4.95; 95% CI [1.88–13.02]) and 30s-STS (HR 2.34; 95% CI [1.14–4.83]).

Patients who improved the above mentioned measures of physical performance had a significantly better survival compared with patients who did not complete 12 months of exercise (6MWT (HR 2.16; 95% CI [1.08–4.32]), handgrip strength right (HR 3,13; 95% CI [1.35–7.26]), handgrip strength left (HR 4.19; 95% CI [1.54–11.36]), IQS left (HR 2.24; 95% CI [1.14–4.39]) and 30s-STS (HR 2.34; 95% CI [1.14–4.83]).

### Multivariate Cox regression analyses

Multivariate analyses were adjusted for age, sex, comorbidity, eGFR, time on dialysis and time as transplanted. The group that improved handgrip strength right (HR 2.66; 95% CI [1.07–6.59]) and handgrip strength left (HR 2.99; 95% CI [1.11–8.07]) by at least 5% had a significantly better survival compared with the group that deteriorated by at least 5%.

The group that improved the 6MWT (HR 2.88; 95% CI [1.41–5.88]), handgrip strength right (HR 4.44; 95% CI [1.79–10.98]), handgrip strength left (HR 5.12; 95% CI [1.83–14.33]), functional reach (HR 3.69; 95% CI [1.82–7.48]), ISQ right (HR 2.86; 95% CI [1.43–5.72]), ISQ left (HR 3.62; 95% CI [1.75–7.48]) and 30s-STS (HR 3.44; 95% CI [1.66–7.11]). by at least 5% had a significantly better survival compared with the group that did not complete 12 months of exercise training.

There were no significant differences in survival between the group that improved by at least 5% and the group that maintained their physical performance in any of the tests.

## Discussion

This post-hoc observational analysis of 151 patients who participated in the RENEXC trial examined the effects of physical performance at baseline and after 12 months of exercise training on survival in patients with CKD stages 3–5 without KRT after a median period of 60 months. Different aspects of physical performance were tested comprising aerobic endurance, handgrip strength, proximal muscle strength in the legs, strength and muscular endurance in the legs and balance.

Aerobic endurance and the 30s-STS at baseline were both independently associated with better survival after adjustment for confounders.

Our findings agree with results from previous observational studies showing that aerobic endurance predicts survival in patients at different stages of CKD and on KRT [8, 12–14, 33]. Several previous studies have used the 6MWT, which is a complex measure of both muscle strength, balance, and muscular and cardiorespiratory endurance, to measure physical performance. In a systematic review of the relationship between survival and physical performance in people with CKD without KRT, MacKinnon et al. found 6 studies with objective measures of physical performance. 6MWT, gait speed and Timed up and go were used and were all associated with a significant reduction in mortality [34], inferring that aerobic endurance measured as walking distance or speed is a useful measure to predict survival in these patients. In the EXCITE trial in people on dialysis who did not partake in the exercise training trial, either because they declined to participate or were deemed ineligible, severe problems with ambulation were associated with higher mortality [14].

After 12 months of exercise training univariate analyses showed that patients with an improved walking distance, 30s-STS and handgrip strength in both hands had a better survival compared with patients who deteriorated. After adjustment for confounders, improved handgrip strength in both hands compared with deterioration was the only test that was significantly associated with survival.

In both the univariate and multivariate analyses, the patients who improved had a better survival compared with patients who did not complete the study, these findings were consistent for all tested aspects of physical performance.

It is noteworthy, that there was no significant difference between patients who improved their physical performance and patients who maintained their physical performance in any of the tests.

In the present study, after adjustment for confounders, walking distance was not associated with a survival benefit for improvers compared with deteriorators, but showed a significant survival benefit for improvers compared with non-completers. Of all patients included about 23% did not complete for various reasons. Compared with completers who improved their physical performance, the non-completers had a lower survival related to all the tests of physical performance. The RENEXC study included old and frail patients to study the effects of exercise training in a real-life situation. For patients in the Swedish Renal Registry’s CKD section, recording patients with CKD stages 3–5 not on KRT, annual mortality was about 13% and start of KRT about 6%. Thus, the proportion of non-completers, 23%, is similar to the Swedish Renal Registry’s real-life data on mortality and start of KRT.

Furthermore, there was no survival benefit for the improvers compared with the group who maintained their physical performance in this study. This is an important finding, as previous studies have shown a gradual deterioration of physical performance with declining GFR [1]. Consequently, the present results could be interpreted that exercise training is associated with a survival benefit for patients with CKD not on KRT, who are able to follow through and complete 12 months of exercise training even if they do not improve. Most likely there are non-accounted for factors affecting general health, which contribute to an inability to complete exercise training and most importantly to an increased risk of mortality for the non-completers.

It is of interest that the group with improved hand grip strength showed better survival compared with the group that deteriorated. Especially as handgrip strength was the only test that was unchanged in the whole group, while all other measures of physical performance improved. These findings imply that exercise training does not primarily affect handgrip strength and that handgrip strength is not a useful method to measure effects of exercise training. Handgrip strength is a measure of distal muscle strength in the arms and hands and correlates with nutritional status [35] and has been found to deteriorate as kidney function declines [36, 37]. In one study, patients with CKD stages 4–5 were shown to have poorer handgrip strength compared with patients with CKD stages 2–3 [38]. Another study found that handgrip strength was better preserved than strength in the lower extremity in patients with CKD stages 2–4 [12]. A study in patients at the start of dialysis showed that better handgrip strength was associated with increased survival [39]. These results were confirmed in a study in patients in CKD stages 3–5 [40]. The results in the literature are conflicting concerning handgrip strength as a predictor of mortality. Handgrip strength is easy to measure and is regarded as a good measure of nutritional status and general health. Deterioration of handgrip strength is regarded as a warning sign, but most likely reflects changes in general health including nutritional status, sarcopenia, degree of inflammation, rather than a worsening of physical performance which is most likely a secondary phenomenon.

This study has some weaknesses. Firstly, we cannot exclude reverse causality in which patients with a higher baseline physical performance have a better general health and thus a lower incidence of mortality; and that patients who are able to complete 12 months of exercise training are healthier and therefore have a lower mortality than patients who are unable to persevere with exercise training for 12 months. Secondly, the group of patients is relatively small and there is no non-exercising control group for comparison. Thus, making it difficult to differentiate whether improved physical performance through exercise improves survival or whether it is easier for healthier individuals to improve their physical performance and therefore have a lower risk of death. Due to small numbers in the present study, chance findings cannot be excluded, and it is possible that the present study did not have enough power to detect real differences in mortality. The clinical significance of exercise training on survival in patients with CKD compared with sedentary controls remains to be elucidated. Future registry-based studies could elucidate this question.

This study has several strengths, such as a relatively long intervention period and follow-up and that a battery of objective methods was used to measure physical performance. There are very few missing data or patients lost to follow-up. In addition, broad inclusion criteria were deliberately used in RENEXC in order to obtain as representative a group of patients as possible.

## Conclusions

Walking distance and muscular strength and endurance in the legs were independently associated with survival in people with CKD stages 3–5 without KRT. Improved walking distance, balance, muscular strength and endurance in the legs and handgrip strength after completing 12 months of exercise training, was associated with better survival compared with non-completers. There was no difference in survival between the completers who improved and the completers who maintained their physical performance.

In this study better physical performance at baseline and the ability to complete 12 months of exercise training conferred survival benefits. Probably a higher level of physical performance is one of several factors affecting survival. These factors require elucidation in future studies.

## Data Availability

Raw data for the datasets in the RENEXC trial are not publicly available to preserve individuals’ privacy under the European General Data Protection Regulation. All data is stored on a locked server at Skåne University Hospital and is available on special request after deidentification of individual data.

## Abbreviations

CKD: Chronic kidney disease
KRT: Kidney replacement therapy
eGFR: Estimated glomerular filtration rate
6MWT: 6-minute walk test
30s-STS: 30-seconds sit-to stand
IQS: Isometric quadriceps strength
HR: Hazard ratio
CI: Confidence interval
